# Neuromuscular Adaptation to Varying Drop Jump Heights: A Muscle Synergy Approach in Elite Handball Players

**DOI:** 10.5114/jhk/217230

**Published:** 2026-04-02

**Authors:** Jinwei Zhao, Kinga Łosińska, Luyao Chen, Piotr Aschenbrenner

**Affiliations:** 1 Department of Physical Education, Gdansk University of Physical Education and Sport, Gdansk, Poland.; 2 Department of Physical Education, Zhengzhou University of Light Industry, Zhengzhou, China.; 3 Faculty of Physical Education, Harbin Sport University, Harbin, China.

**Keywords:** electromyography, motor primitives, single-leg rebound, sport-specific neuromechanics, injury prevention strategies

## Abstract

Rapid single-leg landings and rebounds are central to high-performance handball, yet little is known about neuromuscular coordination adaptations to changing mechanical demands under such conditions. This study examined how different landing heights would modulate lower limb muscle synergy patterns in elite male handball players performing single-leg drop jumps. Twenty professional athletes executed rebound jumps from five standardized heights (0.15–0.75 m). Kinematic data, ground reaction forces, and surface electromyography (sEMG) from seven lower limb muscles were collected and synchronized. Muscle synergies were extracted using non-negative matrix factorization (NMF), while the temporal characteristics of activation patterns were analyzed with SPM1d. Across landing heights, synergy dimensionality remained stable while both muscle weightings and phase-specific activation (pre-activation, buffering, propulsion) were systematically modulated. These height-dependent adjustments were consistent with redistribution within existing motor modules rather than isolated muscle-specific changes. Practically, programming unilateral plyometrics at ~0.60 m may elicit the most pronounced, yet controlled, adaptation of braking and push-off strategies relevant to impact-force management.

## Introduction

Drop jumps are a well-established form of stretch-shortening cycle (SSC) training, commonly utilized to enhance lower limb power and explosive strength in athletic populations (Pedley et al., 2017). In this exercise, the stretch phase begins as the athlete descends from a fixed height, eliciting anticipatory muscle activation to absorb ground impact. This pre-activation phase, primarily eccentric in nature, prepares the neuromuscular system for rapid energy storage (Ruan and Li, 2010). Upon ground contact, the buffering phase begins when elastic elements of the muscle-tendon complex are temporarily loaded (Kosaka et al., 2023). This is followed by the propulsion phase, where concentric contraction of the lower limb extensors drives upward movement (Aboodarda et al., 2013). Unilateral drop jumps are particularly relevant in sports such as handball, where athletes frequently perform single-leg landings after jumps, throws, or dynamic defensive actions.

As a plyometric modality, drop jump training has been shown to improve vertical power, jumping performance, and neuromuscular coordination (Alkjaer et al., 2013; Booth and Orr, 2016). Among the key external variables influencing training efficacy and biomechanical risk, landing height is considered a primary modulator of muscle activation and mechanical load (Peng et al., 2023). Low drop heights optimize explosive responses with minimal joint stress, while moderate heights maximize pre-stretch potential and force output (Bi et al., 2024; Winterholler et al., 2008). Conversely, excessive landing heights may increase joint contact forces and delay muscle-tendon transitions, elevating injury risk (Phillips and Flanagan, 2015). Recent evidence from EMG-guided SSC training in elite badminton players demonstrates that real-time electromyographic feedback can enhance neuromechanical adaptations, including improvements in reactive strength index and EMG latency, thereby optimizing SSC efficiency in sport-specific contexts (Prończuk et al., 2025).

Importantly, the neuromuscular response to drop jumps is not governed by isolated muscle contractions but by coordinated patterns of muscle activation, known as muscle synergies (Hirayama et al., 2017; Rana et al., 2015). According to the muscle synergy theory, the central nervous system organizes complex motor tasks by activating a limited number of functional modules, or synergies, which can be flexibly reconfigured depending on the task demands (Saito et al., 2022; Ting and McKay, 2007). These synergies are hypothesized to be encoded at the brainstem or spinal level and modulated by cortical input. Emerging evidence suggests that cortical modulation can be enhanced through neurofeedback training, with studies showing that EEG-based biofeedback protocols improve cognitive and motor performance in elite athletes, including enhanced reaction times and neuromuscular coordination (Skalski et al., 2024; Skalski et al., 2025). Moreover, optimized environmental conditions, such as normoxia versus normobaric hypoxia, can differentially affect neuromuscular responses and reaction times during biofeedback training (Prończuk et al., 2024). Advances in surface electromyography (sEMG) and non-negative matrix factorization (NMF) have enabled researchers to extract these motor modules and identify both spatial (muscle weighting) and temporal (activation timing) characteristics (Duan et al., 2019; Meattini et al., 2023; Santuz et al., 2017). However, the reliability and validity of synergy extraction depend critically on appropriate EMG normalization and assessment methods, as systematic reviews have highlighted the variability in normalization techniques and their impact on muscle activity interpretation across healthy and clinical populations (Skalski et al., 2025a).

Understanding how muscle synergies respond to progressive changes in external loading (e.g., landing height) is crucial for optimizing training intensity, improving sport-specific movement control, and preventing injury. In particular, elite handball players are exposed to repetitive, asymmetrical, single-leg landings under high loads during match play. These scenarios require fine-tuned neuromuscular coordination to stabilize the lower limb and generate immediate rebound forces. Yet, few studies have examined how such athletes adapt their synergistic patterns across varying landing conditions using synchronized sEMG, force plates, and motion capture systems (Fu et al., 2025).

Prior research has shown that different populations such as Latin dancers or patients with chronic ankle instability exhibit task-specific modulation of muscle activity in response to external perturbations (Akuzawa et al., 2023; Gao et al., 2024; Jie et al., 2024). These adaptations are reflected in both the structure and timing of synergy activation, which may shift based on biomechanical demands. However, investigations focused on healthy elite populations using quantitative synergy extraction and time-series analysis remain limited.

We focused on unilateral landings because single-leg deceleration and immediate re-acceleration are pervasive in handball match play, amplifying frontal-plane control demands and asymmetrical stiffness regulation compared to bilateral tasks. The chosen heights (0.15–0.75 m) span the range routinely used in elite plyometric programming, from low-risk technical exposures to demanding eccentric-concentric transitions that substantially alter joint angles, ground reaction forces profiles, and muscle activation timing. Despite the central role of such tasks in handball, it remains unclear whether progressively higher impacts reorganize synergy composition and timing while preserving global modular dimensionality. We therefore aimed to quantify how landing height would modulate spatial (muscle weightings) and temporal (primitives) features of synergies across pre-activation, buffering, and propulsion phases.

Therefore, this study aimed to investigate how varying landing heights would affect lower limb muscle synergies during unilateral drop jumps in elite male handball players, using a combined methodological framework of sEMG, NMF, kinematics, and force plate analysis. Based on previous findings, we hypothesized that the number of muscle synergies would remain consistent across conditions, but that muscle weighting (motor modules) and activation timing (motor primitives) would vary systematically with height, particularly across the pre-activation, buffering, and propulsion phases. The findings will deepen our understanding of neuromuscular strategies underlying single-leg landings in sport-specific contexts and provide biomechanical insight for injury prevention and performance optimization in elite team sport athletes.

## Methods

### Participants Selection

The required sample size for this study was calculated using G*Power software (version 3.1.9.7) from Heinrich-Heine-Universität Düsseldorf, Germany. With a significance level (α) of 0.05 and a target statistical power of 80%, the analysis determined that a minimum of 13 participants was necessary. Consequently, 20 elite male handball players from the Polish Superliga (top professional division) were recruited for the study.

The participants were aged between 20 and 30 years, with an average body height of 188.2 ± 6.1 cm, body mass of 88.0 ± 8.0 kg, and a body mass index (BMI) ranging from 24.5 to 26.5. All participants had a minimum of five years of continuous professional training experience at the elite level, including regular participation in national league matches and structured strength and conditioning programs. All athletes were right-leg dominant and actively competed during the study period.

### Participant Background

All athletes reported regular exposure to unilateral plyometrics and single-leg landings in their weekly strength and conditioning routines as part of elite handball preparation (>5 years of elite training), ensuring task familiarity.

Inclusion criteria were as follows: absence of neuromuscular or musculoskeletal disorders, no head or spinal cord injuries within the last six months, no history of lower limb injuries in the past year, and no engagement in rehabilitation programs during the testing period. Prior to participation, each athlete received a comprehensive explanation of the study's aims and procedures and provided written informed consent.

This study was conducted following the principles of the Declaration of Helsinki and approved by the Institutional Review Board of the Zhengzhou University of Light Industry, Zhengzhou, China (protocol code: 20240628, approval date: 28 June 2024).

### Experimental Procedure

This study was conducted under controlled laboratory conditions to closely simulate the neuromuscular and biomechanical demands experienced by elite male handball players during real-game scenarios, specifically those involving rapid deceleration, single-leg stabilization, and explosive rebound characteristics of high-intensity defensive or offensive actions.

To assess muscle coordination during single-leg drop jumps from different heights, we used an integrated biomechanical analysis setup composed of high-resolution motion capture, force plate recording, and synchronized surface electromyography (sEMG) acquisition. All data streams were synchronized using Qualisys Track Manager to ensure precise alignment across mechanical and neuromuscular domains.

### Motion Capture and Kinematics

Three-dimensional kinematic data were recorded using an 8-camera Qualisys 600-series motion capture system, sampling at 200 Hz, which tracked reflective markers placed according to the CGM (Conventional Gait Model) lower limb marker set. Marker placement included the anterior superior iliac spines, the posterior superior iliac spines, the lateral and medial femoral epicondyles, the malleoli, the heel, the first to fifth metatarsal heads, and tibial clusters.

To maintain ecological validity relevant to handball performance, all participants wore standardized indoor handball footwear selected for consistency in sole structure, cushioning, medial-lateral stability, and energy return properties. These controlled for intersubject variability in landing force absorption and allowed for safe execution of maximal-effort jumps under repetitive load conditions.

### Ground Reaction Forces

Vertical and multi-axial ground reaction forces (GRFs) were collected using a three-dimensional AMTI force plate (40 cm × 60 cm, 100 Hz), embedded in the laboratory floor. The force plate data were used to delineate key phases of the drop jump and to calculate landing impact profiles relative to drop height.

From vertical GRFs we derived:

– GRF_peak_ (maximal vertical GRF during buffering), normalized to body mass (N/kg);
– time-to-peak (TTP) measured from initial contact (vertical GRF > 5 N) to GRF_peak_ (ms);
– landing impulse calculated as ∫GRF dt from initial contact to the COM nadir, normalized to body mass (N·s/kg);
– rebound jump height computed from flight time as h = g · t^2^* / 8* (with g = 9.81 m·s⁻^2^);
– braking force for take-off defined as the peak negative anteroposterior GRF during the late stance prior to propulsion, normalized to body mass (N/kg).

### Electromyography (EMG) Acquisition

Surface EMG signals were acquired from the dominant leg (right) using a 16-channel Delsys Trigno™ wireless sEMG system, sampling at 2000 Hz. Skin preparation followed SENIAM guidelines: the electrode sites were shaved, abraded, and cleaned with 70% isopropyl alcohol to minimize impedance and reduce motion artifacts. EMG sensors were placed over the following muscle bellies, aligned with fiber orientation: rectus femoris (RF), vastus medialis (VM), vastus lateralis (VL), semitendinosus (ST), biceps femoris (BF), medial gastrocnemius (GM), lateral gastrocnemius (GL).

The placement was based on anatomical palpation and ultrasound-guided positioning where applicable.

### Drop Jump Protocol

Following a standardized dynamic warm-up (10 min, including mobility, balance, and submaximal plyometric drills), participants executed single-leg rebound drop jumps from five progressively increasing heights: 0.15 m, 0.30 m, 0.45 m, 0.60 m, and 0.75 m (Wang et al., 2025). These heights were chosen to reflect typical ranges used in elite plyometric and eccentric loading programs in team sports (Table 1).

**Table 1:** The Effect of different landing heights on the non-negative matrix factorization simplification variables of lower limb muscles in single-leg drop jumps (n = 20).

Reduction Variable	0.15 m (Mean ± SD)	0.30 m (Mean ± SD)	0.45 m (Mean ± SD)	0.60 m (Mean ± SD)	0.75 m (Mean ± SD)	F	*p*	η^2^
Minimum number of synergies	3.05 ± 0.58	3.14 ± 0.47	3.27 ± 0.49	3.27 ± 0.46	3.00 ± 0.53	1.01	0.404	0.37
R2(%)	91 ± 16	89 ± 16	92 ± 15	88 ± 14	87 ± 13	2.43	0.074	0.28

Note: The effect size indicator η2 is defined as follows: 0.01 indicates a small effect, 0.06 indicates a medium effect, and 0.14 indicates a large effect

Each athlete performed three successful trials per height, with 30 s of rest between subsequent repetitions to minimize fatigue. The jump protocol was as follows: first, the participant stood on the plate with the dominant leg slightly in front, the trunk inclined forward, arms relaxed. From this stationary posture, the athlete dropped downward without countermovement, landing on the same leg on the force plate. Upon ground contact, the participant executed an immediate vertical rebound jump, emulating explosive reacceleration after unilateral deceleration, commonly observed in handball actions such as lateral cuts or pivot landings.

Participants were instructed to avoid intense physical activity 24 hours prior to testing to minimize the influence of neuromuscular fatigue. They were instructed to swing their arms naturally to replicate authentic movement patterns, while minimizing excessive compensatory movements. Trials were considered valid if full contact was achieved on the force plate and no balance correction occurred using the non-supporting leg. All testing was conducted under professional supervision, with emergency medical support and protective floor mats available.

### Familiarization and Instructions

Participants underwent standardized familiarization: (i) task briefing and two submaximal single-leg drop-downs per height to ensure safe foot placement and posture; (ii) one coached practice trial per height meeting validity criteria. Verbal instructions emphasized a natural arm swing, single-leg landing fully on the force plate, immediate rebound ‘as fast and as high as possible’, and avoidance of the contralateral leg touch-down or balance corrections. Trials were repeated if any criterion was not met.

### Extraction of Muscle Synergies

To investigate the neuromuscular coordination strategies employed by elite handball players during unilateral drop jumps from varying heights, we performed a muscle synergy analysis based on time-normalized surface electromyography (sEMG) data. This approach allows for the identification of underlying motor modules and temporal activation patterns orchestrated by the central nervous system (CNS) to regulate complex lower limb movement during high-impact tasks common in elite team sports.

We used the musclesyneRgies package implemented in R version 4.4.1 to extract muscle synergies from the processed EMG signals. The analysis employed the Gaussian Non-negative Matrix Factorization algorithm, which decomposes the high-dimensional EMG signal matrix VVV into two non-negative matrices: M (motor modules) and P (motor primitives), such that:

V ≈ Vr = M · P

This decomposition allows for interpretation of neuromuscular control in terms of spatial components (matrix M), representing the relative contribution of each muscle to a given synergy, and temporal components (matrix P), reflecting the time-varying activation profile of each synergy across the movement cycle.

In the analysis, variables M and P were iteratively updated, and the procedure was continued until convergence was reached. A strict convergence criterion was established for the last 20 iterations, requiring that the change in the coefficient of determination R^2^ for reconstruction quality be less than 0.01% (Pan et al., 2024).


$$\begin{array}{c} P_{i+1}=P_{i}\frac{M_{i}^{T}V}{M_{i}^{T}V_{i}P_{i}}\\ M_{i+1}=M_{i}\frac{VP_{i+1}^{T}\text{T}}{M_{i}P_{i+1}P_{i+1}\text{T}} \end{array}$$

The input matrix *V* contained 7 rows (one for each monitored muscle) and 100 columns corresponding to normalized time points covering the entire drop jump cycle from pre-activation (150 ms before landing) to the end of the propulsion phase. Normalization allowed valid inter-trial and inter-subject comparison, essential in high-level athletes performing ballistic actions with small individual variation.

To enhance convergence robustness, we used the Expectation-Maximization (EM) algorithm, which iteratively updated matrices M and P until the coefficient of determination R2 between the reconstructed and original matrix stabilized. The convergence criterion was defined as a change in R2 less than 0.01% over the final 20 iterations, ensuring reliable model performance (Santuz et al., 2017) (Table 1).

For the dimensionality estimation, we factorized EMG with ranks k = 1…6 and selected the minimum k at which reconstruction accuracy R^2^ approached a plateau (ΔR^2^ < 1% with k+1) across participants, indicating no practically meaningful gain from additional components. This decision was made prior to inferential statistics.

For interpretability and classification of synergy structures, we implemented K-means clustering across participants to group similar synergies. The Mean Squared Error (MSE) served as the clustering cost function. Clustering was performed after fixing k to align homologous synergies across subjects; MSE < 5 was used only as a cohesion/quality threshold, not to determine k. Muscles with a relative weight greater than 0.30 were classified as primary contributors to the given synergy (highlighted in Figures 2–4).

**Figure 1:**
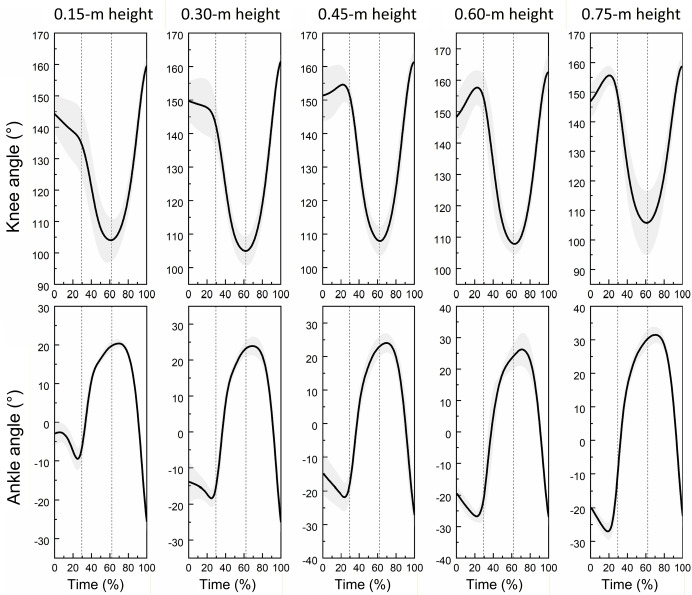
Curves illustrating changes in knee and ankle joint angles of the lower limb during single-leg drop jumps at different landing heights (n = 20). * Figure caption: The vertical dashed lines in the graph divide the movement into the pre-activation phase, the buffering phase, and the extension phase. In the ankle joint angle, positive values indicate dorsiflexion, and negative values indicate plantarflexion

**Figure 2:**
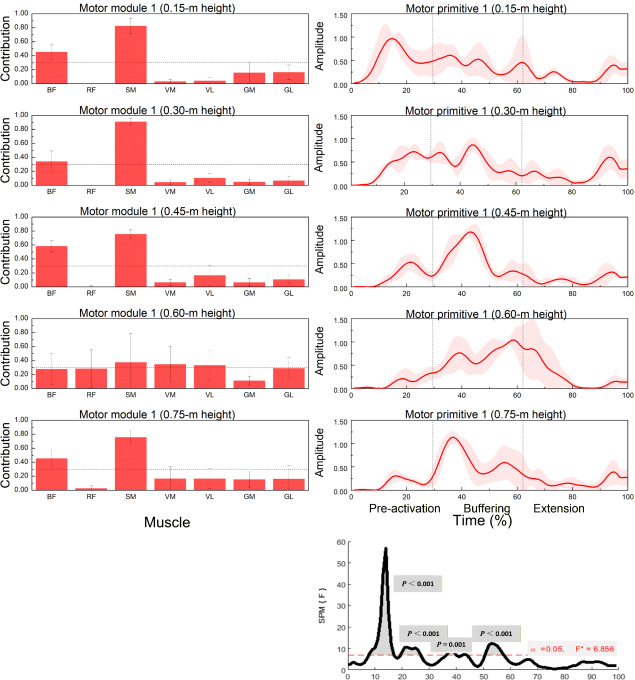
Muscle synergy 1 (posterior-chain dominant): module weights (M) and motor primitive (P1) across landing heights during single-leg drop jumps (n = 20). *Left panels: normalized module weights (M) (mean ± SD) for the BF, RF, SM, VM, VL, GM, and BL muscles at 0.15, 0.30, 0.45, 0.60 and 0.75 m; dotted horizontal line marks the 0.30 contribution threshold used to designate primary contributors (Methods). Right panels: motor primitive P1 (solid line = mean; shaded area = ± SD) over a time-normalized movement cycle (0–100%). Vertical dotted lines indicate phase boundaries based on GRF/COM: pre-activation (−150 ms to IC), buffering (IC to COM nadir), propulsion (COM nadir to TO). Bottom-right: SPM1d{F} across heights; red dashed line = critical F at α = 0.05; grey boxes mark intervals with p < 0.05. Primitives = temporal activations (P); module weights = spatial loadings (M). Abbreviations: BF: Biceps femoris; RF: Rectus femoris; SM: Semitendinosus; VM: Vastus medialis; VL: Vastus lateralis; GM: Gastrocnemius medialis; BL: Gastrocnemius lateralis; IC: initial contact; TO: toe-off; COM: center of mass.

**Figure 3:**
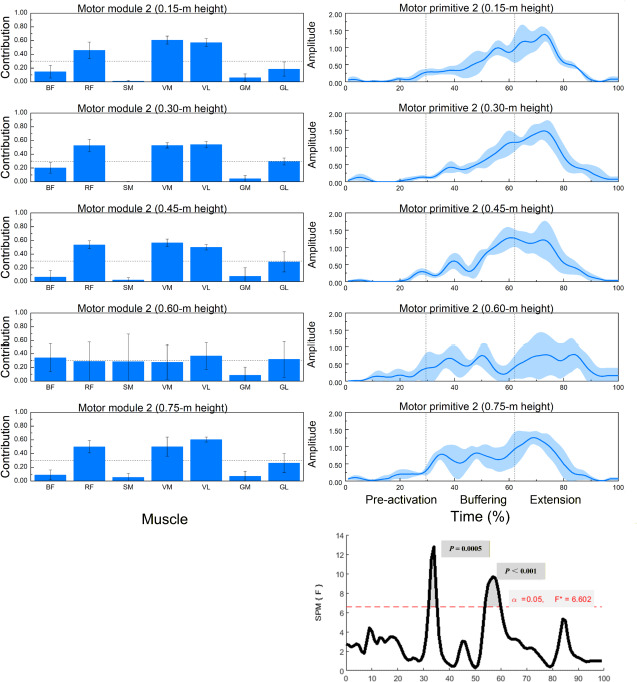
Muscle synergy 2 (anterior-thigh dominant): module weights (M) and motor primitive (P2) across landing heights during single-leg drop jumps (n = 20). * Left panels: normalized module weights (M) (mean ± SD) for the BF, RF, SM, VM, VL, GM, and BL muscles at all heights; dotted horizontal line = 0.30 primary-contributor threshold. Right panels: motor primitive P2 (mean ± SD) over the 0–100% cycle with phase boundaries (IC, COM nadir, TO) delineating pre-activation, buffering, propulsion. Bottom-right: SPM1d{F} across heights with a red dashed α = 0.05 line and highlighted p < 0.05 intervals. Primitives = P (temporal); module weights = M (spatial). Abbreviations: as in Figure 2

**Figure 4:**
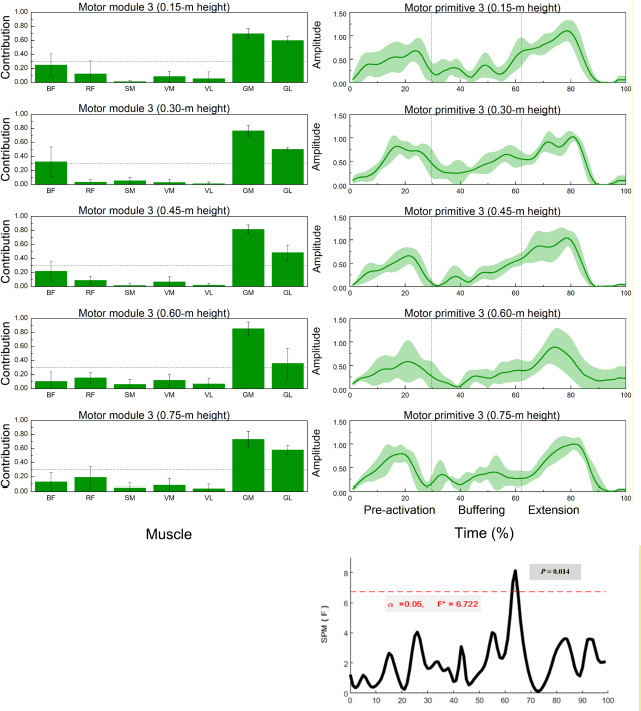
Muscle synergy 3 (gastrocnemius-dominant): module weights (M) and motor primitive (P3) across landing heights during single-leg drop jumps (n = 20). * Left panels: normalized module weights (M) (mean ± SD) for the BF, RF, SM, VM, VL, GM, and BL muscles across heights; dotted line = 0.30 threshold for primary contributors. Right panels: motor primitive P3 (mean ± SD) over the 0–100% cycle with phase boundaries (IC, COM nadir, TO) labelled pre-activation, buffering, propulsion. Bottom-right: SPM1d{F} across heights; red dashed line = α = 0.05 critical F; significant intervals annotated (p < 0.05). Primitives = P; module weights = M. Abbreviations: as in Figure 2

To link the synergy patterns with functional phases of movement, we segmented the single-leg drop jump into three biomechanically distinct intervals (based on GRF and center-of-mass kinematics): (1) pre-activation phase: from 150 ms before the initial contact to GRF > 5 N, (2) buffering phase: from the initial contact to the lowest center-of-mass vertical displacement, (3) propulsion phase: from the lowest point to the moment of the toe-off (GRF < 5 N).

These phase definitions were aligned with Hollville et al. (2019) and reflected real sport-specific motor demands in handball: rapid deceleration after jump shots, reactive changes of direction, and single-leg stabilization under asymmetrical load conditions.

Primitive 1–3 denoted the time-series activation profiles P of the extracted synergies (temporal components), whereas “module weights” denoted the corresponding spatial loadings M (muscle contributions). The abbreviations used were as follows: RF, VM, VL, ST, BF, GM, GL; GRF, GRF_peak_, Impulse, TTP, AP, ML, COM, IC, TO; NMF, SPM1d, EM (algo.), MSE, CGM.

Muscle synergy analysis provides a reductionist yet powerful method to understand how elite athletes economize neuromuscular control by activating consistent, modular patterns rather than regulating each muscle individually. In the context of handball, where unilateral explosive actions are fundamental for performance, such analysis reveals underlying neuromechanical adaptations relevant to power output, dynamic stability, and injury prevention.

### Statistical Analysis

Within the inferential framework, after fixing k as described above, we compared spatial module weights (M) across heights using one-way ANOVA within each synergy and muscle, with appropriate post-hoc tests. For temporal primitives (P), we used SPM1d F-tests over the normalized movement cycle to locate height-dependent intervals. ANOVA/SPM1d thus assessed modulation within a fixed modular structure and did not adjudicate the number of synergies.

All statistical analyses were performed to examine both the temporal dynamics and spatial composition of lower limb muscle synergies across five landing heights. For the analysis of one-dimensional time-series data derived from motor primitives, the Statistical Parametric Mapping method (SPM1d) was employed to detect differences in activation profiles over the normalized movement cycle. The F-test implemented in the SPM1d framework was conducted in MATLAB 2021a, with a two-tailed significance threshold set at α = 0.05 (Pataky et al., 2016). This approach allowed for the identification of statistically significant intervals of modulation in motor primitive waveforms, reflecting height-dependent alterations in the temporal structure of synergy activation.

Complementarily, to assess differences in the relative muscle contribution values (i.e., motor modules), standard statistical procedures were conducted using IBM SPSS Statistics 24. Prior to inferential testing, assumptions of normality and homogeneity of variance were verified using the Shapiro-Wilk test and the Levene’s test, respectively. When these assumptions were met, a one-way analysis of variance (ANOVA) was applied to compare muscle weights across landing heights within each extracted synergy. In cases where significant differences were detected, appropriate post hoc procedures were used: the Bonferroni correction for equal variances and Tamhane’s T2 test for unequal variances.

All descriptive data were reported as means with standard deviations (mean ± SD), and the significance level was uniformly set at 0.05. In addition to *p*-values, partial eta-squared (η^2^) values were calculated to estimate the magnitude of observed effects, interpreted as small (η^2^ = 0.01), medium (η^2^ = 0.06), or large (η^2^ ≥ 0.14), in accordance with established guidelines.

This integrated statistical framework provided both point-wise and continuous insights into how neuromuscular coordination patterns adapted to incremental landing demands, supporting a robust interpretation of synergy-based motor control under sport-specific loading conditions in elite handball athletes.

## Results

### Muscle Synergy Dimensionality and Reconstruction Accuracy

The minimum number of synergies was ≈3 across heights (0.15 m: 3.05 ± 0.58; 0.30 m: 3.14 ± 0.47; 0.45 m: 3.27 ± 0.49; 0.60 m: 3.27 ± 0.46; 0.75 m: 3.00 ± 0.53), with no significant height effect (F = 1.01, *p* = 0.404, η^2^ = 0.37). Reconstruction accuracy R^2^ ranged from 87 to 92% (0.15 m: 91 ± 16%; 0.30 m: 89 ± 16%; 0.45 m: 92 ± 15%; 0.60 m: 88 ± 14%; 0.75 m: 87 ± 13%), with a non-significant trend across heights (F = 2.43, *p* = 0.074, η^2^ = 0.28).

Table 1 presents the effect of different landing heights on the simplification variables derived from non-negative matrix factorization (NMF) of lower limb muscle activity during single-leg drop jumps. Across all conditions (0.15 m to 0.75 m), the number of extracted synergies remained close to three. There were no statistically significant differences in the synergy count between heights (F = 1.01, *p* = 0.404), although the effect size η^2^ = 0.37 suggested moderate variability across conditions.

Reconstruction accuracy, expressed as R^2^, ranged from 87% to 92% and showed no significant height-related differences (F = 2.43, *p* = 0.074, η^2^ = 0.28).

### Ground Reaction Forces and Jump Outcomes

GRF_peak_ and the landing impulse increased monotonically across heights, with the steepest rise from 0.45–0.60 m; TTP shortened slightly at 0.60 m, indicating a stiffer braking strategy. Rebound height peaked at 0.60 m and plateaued or decreased at 0.75 m, consistently with a load threshold limiting elastic return. Braking AP force prior to the take-off increased with height and covaried with gastrocnemius-dominant synergy activity (Synergy 3). Corresponding descriptive statistics are summarized in Table S2 (mean ± SD; forces normalized to body mass where applicable).

### Kinematic Joint Angle Patterns

Figure 1 shows the average profiles of knee and ankle joint angles across the drop jump cycle, segmented into three phases: pre-activation, buffering, and propulsion. Increased dorsiflexion and knee flexion were observed at higher landing heights, particularly at 0.60 m and 0.75 m.

### Synergistic Muscle Activation Patterns

Height-dependent reweighting within fixed modules was confirmed by significant between-height effects in several muscles across Synergy 1–3 (Table 2; multiple F-tests with *p* < 0.05 and medium-to-large η^2^).

**Table 2:** Muscle synergy contribution by height (n = 20).

Synergy	Muscle	0.15 m	0.30 m	0.45 m	0.60 m	0.75 m	F	*p*	η^2^
1	Biceps Femoris	0.46 ± 0.11a	0.34 ± 0.15b	0.59 ± 0.08 a	0.28 ± 0.22b	0.46 ± 0.13 a	3.969	0.013	0.388
1	Rectus Femoris	0.01 ± 0.01b	0.01 ± 0.01b	0.01 ± 0.01b	0.29 ± 0.27a	0.03 ± 0.04b	6.275	0.001	0.501
1	Semitendinosus	0.83 ± 0.11a	0.91 ± 0.05a	0.76 ± 0.06a	0.38 ± 0.41b	0.76 ± 0.10a	6.479	0.001	0.509
1	Vastus Medialis	0.03 ± 0.03b	0.05 ± 0.03b	0.07 ± 0.04b	0.35 ± 0.26a	0.17 ± 0.17a,b	5.324	0.003	0.460
1	Vastus Lateralis	0.04 ± 0.05b	0.11 ± 0.06b	0.17 ± 0.14a,b	0.34 ± 0.22a	0.17 ± 0.14a,b	3.856	0.014	0.382
1	Gastrocnemius Medial	0.16 ± 0.16	0.05 ± 0.04	0.07 ± 0.05	0.11 ± 0.06	0.16 ± 0.11	1.492	0.235	0.193
1	Gastrocnemius Lateral	0.17 ± 0.10 a	0.07 ± 0.06b	0.11 ± 0.06a,b	0.29 ± 0.15a	0.16 ± 0.19a,b	2.902	0.042	0.317
2	Biceps Femoris	0.15 ± 0.09b	0.20 ± 0.08b	0.07 ± 0.09b	0.34 ± 0.20a	0.09 ± 0.07b	5.251	0.003	0.457
2	Rectus Femoris	0.46 ± 0.12	0.53 ± 0.09	0.54 ± 0.06	0.29 ± 0.29	0.50 ± 0.09	2.682	0.055	0.300
2	Semitendinosus	0.01 ± 0.01	0.01 ± 0.01	0.02 ± 0.03	0.28 ± 0.41	0.06 ± 0.06	2.443	0.073	0.281
2	Vastus Medialis	0.61 ± 0.06a	0.53 ± 0.04a,b	0.57 ± 0.05a,b	0.28 ± 0.25c	0.50 ± 0.14b,c	5.668	0.002	0.476
2	Vastus Lateralis	0.57 ± 0.06a	0.54 ± 0.05a,b	0.50 ± 0.04b,c	0.37 ± 0.20c	0.60 ± 0.04a	5.228	0.003	0.455
2	Gastrocnemius Medial	0.06 ± 0.05	0.05 ± 0.05	0.08 ± 0.12	0.09 ± 0.12	0.07 ± 0.07	0.244	0.911	0.038
2	Gastrocnemius Lateral	0.19 ± 0.10	0.29 ± 0.05	0.29 ± 0.15	0.32 ± 0.27	0.26 ± 0.14	0.525	0.718	0.077
3	Biceps Femoris	0.25 ± 0.16	0.33 ± 0.21	0.22 ± 0.13	0.10 ± 0.13	0.13 ± 0.13	2.201	0.098	0.260
3	Rectus Femoris	0.12 ± 0.18	0.03 ± 0.04	0.09 ± 0.06	0.15 ± 0.07	0.19 ± 0.15	1.765	0.167	0.220
3	Semitendinosus	0.01 ± 0.02	0.06 ± 0.05	0.02 ± 0.03	0.06 ± 0.07	0.05 ± 0.07	1.238	0.320	0.165
3	Vastus Medialis	0.09 ± 0.07	0.03 ± 0.05	0.07 ± 0.07	0.12 ± 0.08	0.08 ± 0.09	1.222	0.327	0.164
3	Vastus Lateralis	0.06 ± 0.10	0.01 ± 0.02	0.02 ± 0.02	0.07 ± 0.08	0.04 ± 0.05	0.732	0.579	0.105
3	Gastrocnemius Medial	0.70 ± 0.07a	0.77 ± 0.08a	0.82 ± 0.06a	0.86 ± 0.10a	0.73 ± 0.12a	3.053	0.035	0.328
3	Gastrocnemius Lateral	0.60 ± 0.06a	0.50 ± 0.03b	0.48 ± 0.11b	0.36 ± 0.21c	0.58 ± 0.06a,b	4.087	0.011	0.395

Mean ± SD contribution values of lower limb muscles to three extracted muscle synergies across five landing heights during single-leg drop jumps (n = 20). Significant differences were identified via one-way ANOVA. Letters in the manuscript (a–c) denote significant differences between specific conditions (p < 0.05). Values sharing the same letter within each row are not significantly different from each other (p > 0.05). Values marked with different letters are significantly different (p < 0.05) according to post hoc analysis. Effect sizes: η^2^ ≥ 0.14 (large), η^2^ ≥ 0.06 (medium), η^2^ ≥ 0.01 (small). Normalized module weights (0–1) for each muscle (BF, RF, SM, VM, VL, GM, BL) within synergy 1–3 across landing heights (0.15–0.75 m). F, p, η^2^ report the main effect of height from one-way ANOVA (see Statistical Analysis)

### Muscle Synergy 1: Posterior Chain Dominant Pattern

Figure 2 and Table 2 illustrate the contribution patterns of muscles to synergy 1 across landing heights. The dominant contributors included the biceps femoris (BF) and the semitendinosus (SM). Significant effects of landing height on muscle contribution were observed for the BF (*p* = 0.012), the ST (*p* = 0.008), and also for the rectus femoris (RF), the vastus medialis (VM), the vastus lateralis (VL), and the gastrocnemius lateralis (GL) (*p* < 0.05).

SPM1d analysis revealed significant differences in motor primitives across landing heights within the ranges 8–17%, 19–27%, 36–40%, and 51–57% of the normalized movement cycle.

### Muscle Synergy 2: Quadriceps Dominant Pattern

As shown in Figure 3, synergy 2 was primarily characterized by contributions from the RF, the VM, and the VL. Significant differences in muscle contributions across heights were observed for all three muscles (*p* < 0.01).

SPM1d testing indicated significant variation in the temporal activation of synergy 2 between 32–36% and 53–60% of the movement cycle.

### Muscle Synergy 3 – Gastrocnemius Dominant Pattern

Figure 4 demonstrates that synergy 3 was dominated by the medial (GM) and lateral (GL) heads of the gastrocnemius. Both muscles exhibited significant variation in contribution values across landing heights (GM: *p* = 0.035; GL: *p* = 0.011).

SPM1d analysis showed phase-specific modulation in motor primitive activity between 62 and 65% of the movement cycle.

## Discussion

The primary aim of this study was to investigate the influence of different landing heights on lower limb muscle synergies during single-leg drop jumps in elite male handball players. By applying non-negative matrix factorization (NMF) to surface EMG signals, and analyzing phase-specific modulation using statistical parametric mapping (SPM1d), we explored how neuromuscular coordination patterns adapted across progressive impact conditions. Athletes performed under a highly standardized protocol reflecting game-specific unilateral deceleration and propulsion, typical in elite-level handball (e.g., after cutting, jump shooting, or rebounding from contact).

Our finding of invariant dimensionality with height-dependent re-weighting and timing aligns with modular control accounts in dynamic tasks, where the CNS flexibly retunes existing modules rather than recruits additional ones when external demands increase moderately (Rizzato et al., 2023; Santuz et al., 2017; Ting and McKay, 2007). Within this framework, hamstring-dominant buffering at lower heights and progressive quadriceps/calf involvement at ~0.60m are coherent with known adjustments of joint stiffness and tendon loading during drop landings (Bi et al., 2024; Hollville et al., 2019; Peng et al., 2023; Phillips and Flanagan, 2015).

These results also resonate with prior studies on the phase-specificity of muscle recruitment under varying plyometric loads, emphasizing context-dependent redistribution within consistent modules rather than fundamental changes to synergy structure (Bi et al., 2024; Kipp et al., 2014).

The results confirmed the presence of three robust muscle synergies across all landing heights, indicating a consistent modular organization of neuromuscular control. These findings align with prior research demonstrating that a small set of coordinated motor modules can efficiently regulate complex movements (Rizzato et al., 2023; Ting et al., 2015). Although the number of synergies did not differ significantly, variation in the contribution of specific muscles within each synergy revealed dynamic reorganization in response to increasing biomechanical demands.

### Synergy 1: Posterior Chain Dominant Strategy during Pre-activation and Buffering

The first synergy was primarily composed of the biceps femoris and semitendinosus, muscles integral to eccentric knee control and hip extension. This synergy showed the greatest modulation during the pre-activation and buffering phases, as evidenced by SPM1d results indicating significant waveform differences within 8–57% of the movement cycle. At lower drop heights (0.30–0.45 m), hamstring dominance was most pronounced, potentially reflecting the neuromechanical strategy to minimize joint loading through posterior muscle activation. This is particularly relevant for elite handball players, where rapid landing recovery following offensive or defensive maneuvers requires precise eccentric regulation to decelerate body mass and protect the knee joint.

As drop height increased, the contribution of the posterior thigh muscles diminished, while greater engagement of the rectus femoris, vastus medialis, and vastus lateralis muscles was observed at 0.60 m. This shift may indicate a redistribution of buffering responsibility toward the quadriceps group, as higher impact forces necessitate greater extensor stiffness. Such anterior chain adaptation has been previously linked to alterations in lower limb stiffness and joint coordination under high-load conditions (Kosaka et al., 2023).

The lateral gastrocnemius also showed a significant increase in activation at 0.60 m, suggesting heightened ankle stabilization demands. This finding aligns with previous observations indicating that higher landing forces may lead to a shift from hip-dominant to knee-ankle dominant control strategies in athletes performing single-leg deceleration (Bi et al., 2024; Peng et al., 202).

### Synergy 2: Anterior Chain Activation during Propulsion

The second synergy predominantly involved the rectus femoris, vastus medialis, and vastus lateralis muscles, which are key contributors during the propulsion phase of drop jumps. Although the overall contribution of the rectus femoris remained stable, the vastus medialis and the lateralis exhibited significant modulation across landing heights, particularly peaking at 0.60 m. These results, supported by SPM1d data (significant differences in 32–60% of the cycle), highlight the involvement of the quadriceps in controlling the transition from eccentric braking to concentric push-off.

Given that handball requires repeated single-leg rebounds and explosive vertical acceleration (when contesting high balls or initiating counterattacks), the quadriceps synergy appears critical in maintaining performance efficiency and reducing cumulative joint stress. The stable contribution of the RF across conditions suggests its pivotal role in knee extension, whereas VM and VL appear more sensitive to vertical loading magnitude, likely reflecting adjustments in frontal-plane stabilization and patellofemoral tracking under increased demands.

### Synergy 3: Calf Muscle Engagement in Late Buffering and Take-off

The third synergy included the medial and lateral gastrocnemius muscles, which were especially active during the final buffering and early propulsion phases. The modulation of these muscles, particularly the increased contribution at 0.60 m, suggests their importance in late-phase force redirection. This synergy was significantly altered between 62 and 65% of the jump cycle, consistently with ankle-driven propulsion and stiffness regulation at push-off.

For handball players, whose sport demands high levels of ankle joint control during the landing and the take-off, this synergy is crucial. Adequate activation of the gastrocnemius ensures efficient transfer of stored elastic energy and supports plantarflexion under loading (Kipp et al., 2014). It also contributes to proprioceptive joint stabilization, reducing the risk of ankle inversion, which is a common injury mechanism in handball (Gao et al., 2024; Jie et al., 2024).

### Neuromuscular Adaptability and Sport-Specific Implications

Collectively, the data are consistent with redistribution within a stable set of motor modules, evidenced by an unchanged synergy count and R^2^ alongside significant, phase-specific changes in muscle weightings and primitive waveforms across heights; however, causal claims about neural implementation should be made with caution (Santuz et al., 2017; Ting and McKay, 2007). This supports the concept of modular flexibility within the motor control system, where the invariant synergy number coexists with variable muscle weighting and activation timing. These adjustments reflect the neuromechanically flexibility of elite athletes, who must repeatedly cope with varied external forces during rapid transitions between offensive and defensive tasks. Such neuromuscular adaptability is particularly relevant in handball, a sport characterized by frequent single-leg landings, dynamic reaccelerations, and direction changes.

The findings also emphasize that landing from moderate heights (~0.60 m) elicits the most pronounced muscle reorganization, as previously described for threshold-dependent adjustments in neuromuscular and tendon responses to drop height (Hollville et al., 2019; Peng et al., 2023; Phillips and Flanagan, 2015). This may indicate an optimal stimulus range for eliciting central nervous system adaptations during stretch-shortening cycle tasks.

From a practical standpoint, this height may serve as a benchmark for plyometric training in handball, eliciting sufficient neuromuscular demand without excessive joint stress. It provides a valuable reference for load progression in unilateral eccentric-concentric exercises. The identified synergy shifts can inform targeted interventions to enhance load distribution, movement economy, and injury resilience, particularly through strengthening the posterior chain for deceleration control and the calf complex for late-phase propulsion. These insights may contribute to designing individualized training protocols that better reflect the movement demands of elite handball gameplay.

**Figure 5:**
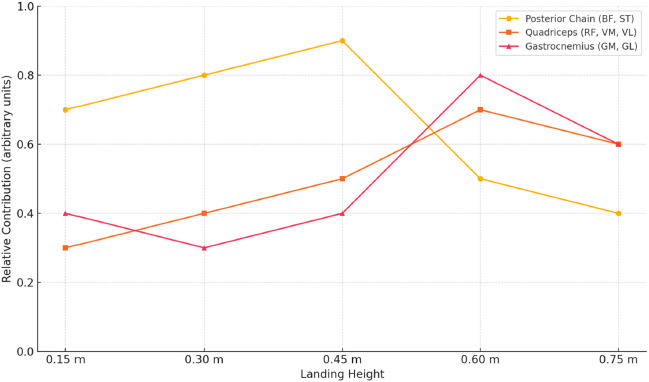
Adaptation of lower limb muscle synergy profiles across increasing landing heights in elite handball players. * Note: The figure illustrates changes in the relative contribution of three major muscle groups (posterior chain: BF, ST; quadriceps: RF, VM, VL; gastrocnemius: GM, BL) across five landing heights during single-leg drop jumps. Posterior chain dominance is most evident at lower heights (0.30–0.45 m), indicating their primary role in eccentric control. As height increases, quadriceps and gastrocnemius contributions rise, peaking at 0.60 m. This pattern reflects a shift toward anterior and distal recruitment strategies, supporting increased stabilization and propulsion demands. The model visualizes neuromuscular adaptation among elite handball athletes to progressively intensified landing stimuli

This neuromuscular shift from posterior to anterior and distal dominance across landing heights is summarized in Figure 5, which illustrates the adaptive synergy modulation observed in elite handball players.

### Limitations and Future Directions

This study focused exclusively on elite male handball players, which limits generalizability to female athletes or less trained populations. Prior research indicates that sex-related differences in neuromuscular strategies may affect landing biomechanics and injury risk (Kipp et al., 2014). Future studies should expand the sample to include female handball players and integrate joint moment analysis or inverse dynamics to provide more comprehensive insight into load distribution.

Furthermore, combining synergy analysis with real-game perturbation tasks or fatigue protocols may enhance ecological validity and better reflect sport-specific constraints. Finally, applying neurophysiological tools such as transcranial magnetic stimulation (TMS) or high-density EMG could help elucidate the cortical or spinal mechanisms underpinning observed synergy modulation.

## Conclusions

This study demonstrated that the modular organization of lower limb muscle coordination in elite male handball players remains stable across various landing heights, with three consistent synergies underpinning single-leg drop jump performance. However, the spatial and temporal structure of individual synergies is dynamically modulated in response to changing biomechanical demands.

At moderate landing heights (~0.60 m), the most pronounced reorganization of muscle contributions was observed, particularly a shift from posterior to anterior and distal muscle dominance. This reflects the neuromuscular system's ability to adjust load distribution and joint stabilization strategies according to the task intensity.

These findings provide valuable insights for performance optimization and injury prevention in high-level team sports. Incorporating height-specific plyometric loading into training may enhance neuromuscular adaptability, improve single-leg force control, and reduce injury risk, particularly in scenarios mimicking high-speed landings and explosive redirection typical of elite handball gameplay.

The 0.60-m height may represent a neuromuscular threshold useful for programming eccentric overloading in single-leg plyometrics for handball.
